# Evaluation of PRRSv specific, maternally derived and induced immune response in Ingelvac PRRSFLEX EU vaccinated piglets in the presence of maternally transferred immunity

**DOI:** 10.1371/journal.pone.0223060

**Published:** 2019-10-02

**Authors:** Christian Kraft, Rimma Hennies, Karla Dreckmann, Marta Noguera, Poul Henning Rathkjen, Michael Gassel, Marcus Gereke

**Affiliations:** 1 Boehringer Ingelheim Veterinary Research Center GmbH & Co. KG., Hanover, Germany; 2 Boehringer Ingelheim Vetmedica GmbH, Ingelheim, Germany; Stanford University School of Medicine, UNITED STATES

## Abstract

In this study, we analyzed PRRS virus (PRRSv) specific lymphocyte function in piglets vaccinated with Ingelvac PRRSFLEX EU^®^ at two and three weeks of age in the presence of homologous maternal immunity. Complete analysis of maternal immunity to PRRSv was evaluated postpartum, as well as passive transfer of antibodies and T cells to the piglet through colostrum intake and before and after challenge with a heterologous PRRSv at ten weeks of age. Maternal-derived antibodies were detected in piglets but declined quickly after weaning. However, vaccinated animals restored PRRSv-specific antibody levels by anamnestic response to vaccination. Cell analysis in colostrum and milk revealed presence of PRRSv-specific immune cells at suckling with higher concentrations found in colostrum than in milk. In addition, colostrum and milk contained PRRSv-specific IgA and IgG that may contribute to protection of newborn piglets. Despite the presence of PRRSv-specific Peripheral Blood Mononuclear cells (PBMCs) in colostrum and milk, no PRRSv-specific cells could be detected from blood of the piglets at one or two weeks of life. Nevertheless, cellular immunity was detectable in pre-challenged piglets up to 7 weeks after vaccination while the non-vaccinated control group showed no interferon (IFN) γ response to PRRSv stimulation. After challenge, all piglets developed a PRRSv-specific IFNγ-response, which was more robust at significantly higher levels in vaccinated animals compared to the primary response to PRRSv in non-vaccinated animals. Cytokine analysis in the lung lumen showed a reduction of pro-inflammatory responses to PRRSv challenge in vaccinated animals, especially reduced interferon (IFN) α levels. In conclusion, vaccination of maternally positive piglets at 2 and 3 weeks of age with Ingelvac PRRSFLEX EU induced a humoral and cellular immune response to PRRSv and provided protection against virulent, heterologous PRRSv challenge.

## Introduction

The porcine reproductive and respiratory syndrome (PRRS) is caused by an enveloped RNA virus affecting pigs worldwide and leading to great economic losses in the swine industry [1]. Infections can occur in pigs of all ages, provoking respiratory syndromes, cyanosis of extremities as well as reproductive failure in sows [2]. Two main PRRS viruses (PRRSv), PRRSv-1 and PRRSv-2 [3, 4] have been described so far, while PRRSv-1 was further divided in several subtypes with unique features [5, 6]. Although similarities exist between and within the PRRSv-1 strains, there is enough diversity that vaccines may not induce sufficient cross-protection against heterologous strains [7]. Genetic diversity of PRRSv is such that all challenge situations in the field could be considered heterologous [8]. Nevertheless, vaccination with a modified life virus (MLV) against PRRSv has been demonstrated as an effective tool to control clinical signs related to infection [9, 10], while killed virus (KV) vaccines, when used for priming, could not induce an effective protection against the clinical outcome of disease [11].

The neonatal piglet is vulnerable to infection by pathogens [12], but protection is acquired by the transfer of maternal immunity through colostrum and milk. The beneficial effect of colostrum intake within the first 24 hours of life has been investigated extensively and proven positive for newborn growth and health status even beyond the suckling period [13] [14] [15]. Maternal antibodies can be found in piglets right after intake of colostrum, but the concentration in serum drops below detection levels within a few weeks after weaning [16]. During this time period, the piglet is vulnerable against infections since protection provided by maternal immunity through colostrum is waned. Interference of maternally derived antibodies (MDA) with vaccination has been reported for different pathogens like Swine influenza A virus [17], Aujeszky’s disease [18] and others. Previous studies in the field with a PRRS type 1 MLV vaccine indicated a detrimental effect on the development of PRRSv-specific piglet immunity at 4 weeks after vaccination [19] and a delayed vaccine effect could only be detected after the MDA had declined. However, no direct conclusion on vaccine efficacy could be determined in this study due to a lack of field challenge. In another field study, vaccination in the presence of MDA showed a reduction of clinical signs after natural exposure of growing pigs to a field strain of the same genotype; however, vaccination did not reduce the number of viremic animals or the level of viremia [20]. In contrast, vaccination with a different PRRS type 1 vaccine at two or three weeks of age in the face of MDA followed by a heterologous field challenge protected vaccinated pigs from clinical signs and viremia [21]. However, vaccine specific immune correlates in the colostrum, milk or blood was not analyzed in detail. A recent publication by Balasch et. al described an effective vaccination in 1-day-old piglets by a MLV PRRSv vaccine in the presence of maternal antibodies with subsequently challenge [22] and demonstrated the potential of MLV vaccines in the presence of maternal antibodies. However, PRRSv specific cellular immune responses were not analyzed.

Since a transfer of immune cells through the placental tissue is blocked [23], cell mediated maternal immunity has to be transferred to the piglet by the colostrum and milk exclusively [24]. The passive transfer of cells from the sow to their offspring has been demonstrated in colostrum [25] [26] and its protective function was specifically reported for *Mycoplasma* subspecies infections [27] [28]. Nevertheless, the role and function of PRRSv specific cell mediated immunity transferred to the piglet is not yet well understood.

In this study we investigated the transfer of PRRSv-specific maternal immunity from the dam to the piglet. Furthermore, we determined the induction of the piglets’ humoral and cellular immunity as well as the efficacy of the Ingelvac PRRSFLEX^®^ EU vaccine in the presence of maternally derived homologous PRRSv-specific immunity. Moreover, the efficacy of vaccination in the presence of MDA was assessed by a controlled, heterologous PRRSv challenge at ten weeks of age.

## Material and methods

### Animals

In total, 26 sows (commercial, mixed-breed, mixed parity and Topigs Norsvin genetics) were obtained from a conventional closed system piglet producing farm in Germany at approx. 60 days of gestation. Eighty nine, clinically healthy piglets born from these sows were included in the study.

The farm was known to be seropositive for PRRSv-1; however, animals were free of an acute PRRSv infection, as none of the control animals were tested positive (determined by qPCR [9]) before challenge.

Experimental procedures were approved by the institutional ethics committee, the Advisory Committee for Animal Experiments (§12 of Law for Animal Experiments, Tierversuchsgesetz–TVG) and the Federal Ministry for Science and Research (reference number BMWF-68.205/0083-II/3b/2013).

### Study outline

All sows of the breeding herd were vaccinated with ReproCyc^®^ PRRS EU (Boehringer Ingelheim Vetmedica GmbH, Germany) in a mass vaccination scheme. Sows scheduled to farrow nine weeks post vaccination were included in the study. Three to four piglets were selected per litter to minimize a litter effect (i.e. depending on litter size every second or third piglet as lined up during suckling). A total of 60 piglets were vaccinated once intramuscularly in the neck with Ingelvac PRRSFLEX^®^ EU (Boehringer Ingelheim Vetmedica GmbH, Germany) at two or three weeks of age according to label instructions, and an additional 29 animals received a mock vaccination with a placebo (Table 1). All study animals were housed in stables appropriate for their breed and age and were kept under similar controlled conditions. In farrowing units under field conditions, the vaccinated animals were housed separately from non-vaccinated, control animals for biosecurity purposes. Piglets were transferred to a controlled laboratory environment (BSL2 conditions) at weaning. As no clinical signs were detected until day of experimental challenge it can be assumed that no concurrent infection with other pathogens could be suspected. At 7–8 weeks post vaccination, piglets received an experimental challenge with a heterologous PRRSv strain. Necropsy was performed ten days post challenge (dpc).

**Table 1:** Group assignment of piglets.

Sow vaccination	Piglet vaccination	No.	Challenge
**ReproCyc PRRS EU**	**PRRSFLEX EU, 2-woa**	**30**	**Yes**
**ReproCyc PRRS EU**	**PRRSFLEX EU, 3-woa**	**30**	**Yes**
**ReproCyc PRRS EU**	**Control**	**29**	**Yes**

### Challenge

With ten weeks of age piglets were challenged with the virulent, low-passage type 1 PRRSv isolate 190136, at a Tissue Culture Infective Dose 50% end-point (TCID_50_) of 8x10^5^ per ml. The challenge isolate was propagated in the MA104 cell line at a low passage to remain its virulence. The challenge inoculum was originally obtained from the lung tissue of a newborn piglet on a farm showing typical reproductive signs of PRRSv (abortions in sows and weakness in newborn piglets) during an outbreak in Lower Saxony, Germany, in April 2004. The challenge isolate and the vaccine strain are heterologous members of type 1 PRRSv, subtype 1, isolated from geographically distinct regions in Germany and exhibiting less than 87% genetic identity within the complete genome (or 88% with the ORF5/ORF7; data not shown). The challenge material was administered intranasally using a nebulizer device with 1 ml per nostril.

### Serology

Blood was collected via jugular venipuncture from the piglets on the day of birth and on days 7, 14, 21, 28, 42, 61 days of life, and the days of challenge and necropsy. The sample volume was adjusted appropriate for the age and weight of the piglet ranging from a maximum of 2 ml to 9 ml per sampling. Blood was processed for serum and used to determine the S/P ratio of antibodies against PRRSv by ELISA using a commercial ELISA kit (Idexx Herd-Check X3 ELISA from IDEXX Laboratories, Inc., Westbrook, Maine, USA) according to the manufacturers’ specifications, or to determine the PRRSv specific IgG and IgA levels by ELISA using a commercial ELISA kit (INgezim PRRS Universal 1.1.PRU.K1 ELISA Kit from INGENASA, Madrid, Spain) modified by using horse radish peroxidase (HRP) conjugated Goat α-porcine IgG Fc and HRP conjugated Goat α-porcine IgA detection antibodies from Bethyl (BETHYL Laboratories, Inc., Montgomery, Tx, USA).

### Necropsy

All piglets were euthanized by electrocution and then exsanguination and necropsied 10 days after challenge. The thoracic and abdominal cavities were exposed and examined for gross lesions. The lung and trachea were removed intact and with a clamp placed across the trachea to prevent blood cross contamination to enable bronchial alveolar lavage fluid (BALF) preparation. Following removal, the lungs of each animal were scored to determine the percentage of lung lesions for each lobe before samples were taken. Multiplied by a lobe-specific correction factor, this gave the score for each lobe. A total lung lesion score was determined for each animal by the summation of percent lung consolidation (lesions) that was observed for each lung lobe. The assessed percentage of lung lobe area with typical lesions was multiplied by the lobe factor (i.e., left apical = 0.05, left cardiac = 0.06, left diaphragmatic = 0.29, right apical = 0.11, right cardiac = 0.10, right diaphragmatic = 0.34, and intermediate = 0.05), and the total weighted lung lesion score was determined.

### Cell purification from colostrum and milk

Colostrum was sampled on the day of farrowing and milk was sampled one day after farrowing by milking the sow. Samples were collected in 50ml tubes and cells were enriched the same day using routine protocols (layering colostrum or milk over high density Ficoll gradient and centrifugation at 1200g for 10 min in SepMateTM tubes (STEMCELL Technologies Germany GmbH, Köln, Germany). Leukocytes were washed extensively in PBS (no calcium, no magnesium). The pellet was re-suspended in cell culture medium. The cells were counted by using trypane blue and the TC20 counter (Bio-Rad AbD Serotec GmbH, Puchheim, Germany). The IFNγ-ELISPOT was performed by using 2.5x10^5^cells/well for RPMI stimulus and 5x10^5^cells/well for PRRSv restimulation. Final spot forming units (sfu) were adjusted to 1x10^6^ cells/well.

### Peripheral Blood Mononuclear Cell (PBMC) isolation

At 9 weeks of life and at necropsy blood was collected in heparinized tubes and PBMCs was enriched the same day using routine protocols (layering blood over high density Ficoll gradient and centrifugation at 1200g for 10 min in SepMate^TM^ tubes (STEMCELL Technologies Germany GmbH, Köln, Germany). Leukocytes were washed extensively in Phosphate buffered saline (PBS) (no calcium, no magnesium). The pellet was resuspended in cold freezing medium (containing 50% RPMI1640 Medium, 40% FCS and 10% DMSO), dispensed in 2ml cryovials, and transferred to -70°C freezer and after 24h transferred into -150°C freezer.

#### Bronchial alveolar lavage fluid

The respiratory tract including intact lungs and trachea was removed from animals immediately following euthanasia. Forty milliliters of cold PBS was then added through the trachea using a 50 ml syringe (BD, Heidelberg, Germany). The lung tissue was gently massaged by hand and inverted to collect the fluid in 50 ml conical tubes. The BALF was then centrifuged for 10 minutes at 800g at 4°C. The supernatant was collected and stored at -80°C.

#### IFNγ-ELISpot

Several 96 well plates with a polyvinylidene fluoride membrane (Millipore, Billerica, USA) were coated with purified anti-porcine IFNγ antibody (Mabtech, Nacka Strand, Sweden) for at least 12 h. Milk derived cells or PBMCs were thawed, washed twice in RPMI medium, counted using trypane blue, and adjusted to be dispensed to 3x10^5^ cells/well. After extensive washing of the plate containing the coating antibody with PBS, cells were seeded. Cell culture medium was added containing 3 μg/ml of the polyclonal activator ConA (positive control of IFNγ release) or with different concentrations of the antigen (PRRS virus). Wells containing only medium or unstimulated cells served as negative controls. After stimulation for 48 h, plates were washed with water and PBS/0.01% Tween20, and incubated at room temperature for 1–2 h using a detection biotinylated anti-bovine IFNγ antibody (Mabtech, Nacka Strand, Sweden). Subsequently, streptavidin-alkaline phosphatase enzyme (Roche, Mannheim, Germany) was added to the plates (in the dark, for less than 1 h incubation). Finally, NBT and 5-Bromo- 4-Chloro-3-Indolyl Phosphate (Sigma, Munich, Germany) were used as substrate of the alkaline phosphatase. These substrate systems produce an insoluble NBT diformazan end product that is blue to purple in color and can be measured in a plate reader. After extensive washing with running tap water, plates were left overnight to ensure complete drying. Spot counting was performed using the C.T.L. ELISpot reader (CTL, Bonn, Germany). Final sfu were adjusted to 1x10^6^ PBMC.

### Detection of cytokines

Cytokines (IL-1β, TNFα and IFNα) were detected in BALF via ELISA. IL1β and TNFα content were detected by ELISA kits from R&D Systems (R&D Systems Inc., Minneapolis, USA) according to manufacturer’s protocols. IFNα was detected by an in house ELISA with anti-porcine IFNα antibodies and recombinant porcine IFNα as standard by Kingfisher Biotech (Kingfisher Biotech, Inc., Saint Paul, MN, USA). In general, for this technique, 96-well high binding plates were coated with a purified antibody specific for the cytokine of interest and incubated overnight at 4°C. On the next day the plate was blocked with blocking buffer containing bovine serum albumin (BSA) for 1–2 h at room temperature. Afterwards BALF was incubated on the plate for 2 h at room temperature. Wells containing only the dilution buffer instead of the supernatant were used as the blank. Plates were washed several times with a phosphate buffer containing 0.05% Tween20. Then, a detection antibody or a HRP conjugated antibody, binding against the cytokine of interest, were incubated on the plate for 1–2 h at room temperature. After washing, either streptavidin was added for 30min or the plate was directly incubated with 100μl/well of 3,3',5,5'- TMB. After 10–20 min the reaction was stopped by adding 50μl/well of 2N Sulfuric Acid and the plate measured using the BioTek ELISA Reader (BioTek, Bad Friedrichshall, Germany) at a wavelength of 450nm.

### Cell thawing

PBMCs stored frozen in suspension in freezing medium in 1.8 ml cryotubes were placed in a 37°C water bath until nearly thawed, and then diluted in 13 ml of 37°C pre-warmed PBS. The cells were then washed in 5 ml of PBS, counted via TC-20 cell counter (Bio-Rad AbD Serotec GmbH, Puchheim, Germany), and then diluted in RPMI1640 (Gibco, Waltham, MA, USA) containing 10% (v/v) FCS and 100 IU/ml penicillin/0.1 mg/ml streptomycin (Gibco, Waltham, MA, USA) to a concentration of 3x10^6^ live cells/ml.

## Results

### Vaccination with Ingelvac PRRSFLEX^®^ EU overcomes maternally derived PRRSv-specific antibody interference and protects piglets from heterologous PRRS virus challenge

**Fig 1:**
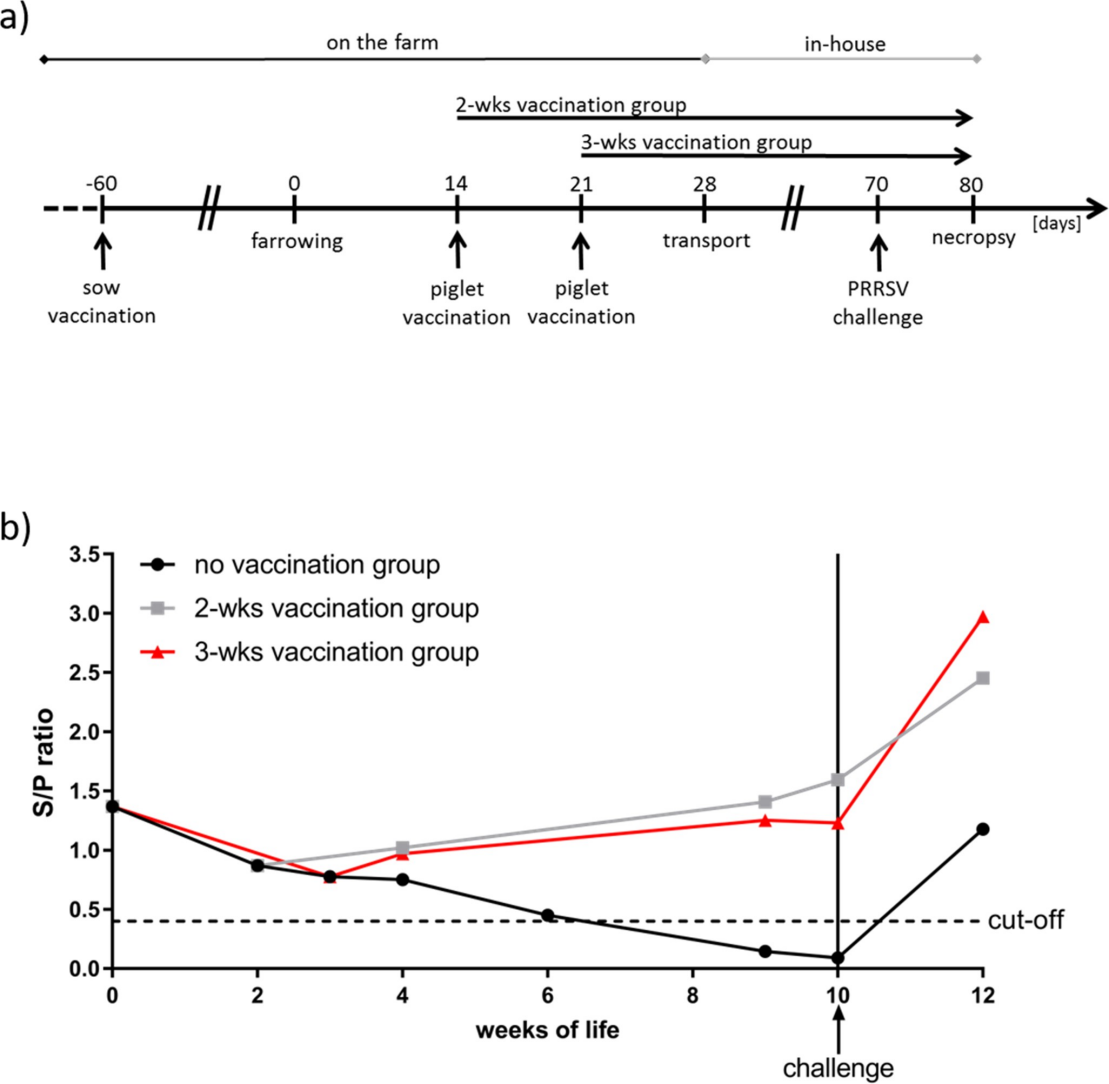
**a) Graphical scheme of the experimental study design. b) PRSSv-specific antibody levels in piglets over time.** Sera from piglets were analyzed on a weekly basis for PRRSSV-specific antibodies by Idexx-Herd check X3 ELISA. Single values were calculated as sample to positive control ratio (S/P) and were depicted as mean values within the groups (no vaccination group, 2-wks vaccination group, 3-wks vaccination group). The first samples in week 0 were taken after colostrum intake. Significant differences were calculated by Mann-Whitney-test and were indicated in the graph. Significant differences of ****p ≥ 0.0001 at 9 weeks between no vaccination group vs. 2-wks and 3-wks vaccination group; **p = 0.0036 at 10 weeks between no vaccination group vs. 2-wks and 3-wks vaccination group; ****p ≥ 0.0001 at 12 weeks between no vaccination group vs. 2-wks and 3-wks vaccination group and *p = 0,0426 at 12 weeks between 2-wks vs. 3-wks vaccination group.

This study was designed to investigate PRRSv-specific maternally derived antibody (MDA) interference when vaccinating sows with a homologous PRRSv MLV and to investigate if piglet vaccination can overcome maternal immunity and protect the piglets from a heterologous PRRSv challenge. The experimental scheme is described in Fig 1A.

At different time points blood samples collected from piglets were serologically analyzed for a PRRSv-specific humoral immune response (Fig 1B). All piglets were sero-positive for PRRSv-specific antibodies on the day of birth due to colostrum intake before blood sampling. Antibody levels were comparable in all groups until weaning at 4 weeks of age with no significant difference between all three groups. However, in the non-vaccinated control group the maternal antibodies declined over time after weaning to a serologic PRRSv-negative status at nine to ten weeks of age. These results suggest that vaccination at 2 or 3 weeks of life was conducted in the full presence of PRRSv-specific MDA. In contrast to the non-vaccinated control group both vaccinated groups showed a constant level of anti-PRRSv antibodies in their blood indicating that acquired humoral immunity through vaccination with Ingelvac PRRSFLEX EU filled the gap of consistently declining maternal antibody levels. At challenge (7–8 weeks post vaccination), vaccinated animals had significant higher PRRSv-specific antibody levels compared to the non-vaccinated control group. After challenge, all groups responded with an increase in PRRSv-specific antibody levels, however, both groups of vaccinated animals (two or three weeks of age) showed a statistically significant increased level of PRRSv-specific antibodies compared to non-vaccinated control animals (*p* ≤ 0.0001; Fig 1B).

**Fig 2:**
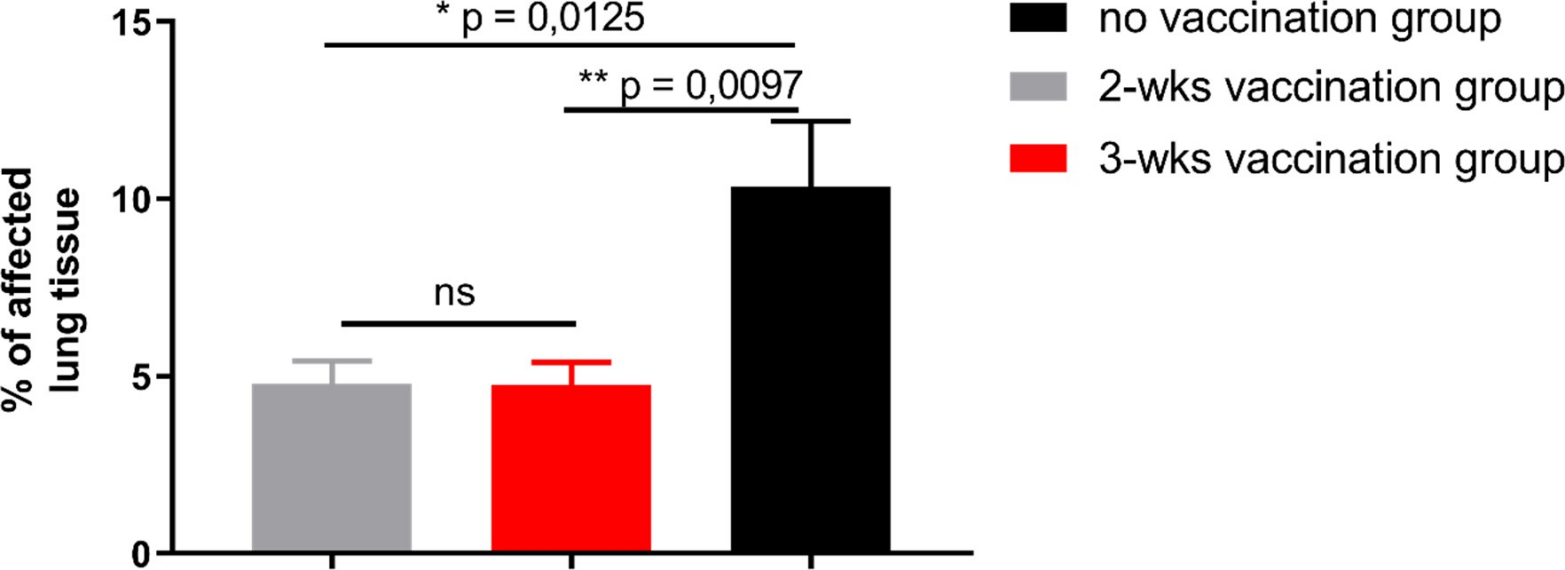
Lung lesion score. Lungs were analyzed 10 days after PRRSv-challenge at necropsy for PRRSv-specific lesions. Data are displayed as mean values (% of affected lung tissue) with SEM within the groups (no vaccination group, 2-wks vaccination group, 3-wks vaccination group). Significant differences were calculated by Mann-Whitney-test and calculated *p*-values were indicated in the graph.

All animals were necropsied 10 days post challenge and the lungs were thoroughly investigated by a certified veterinary pathologist for evidence of lesions indicative of PRRSv infection. The adjusted percentage of affected lung tissue per lobe was determined and the mean lung lesion scores were calculated (Fig 2). All lung lesions were found to be suggestive for a PRRSv infection. No indication was found that other respiratory pathogens were involved in the formation of lung lesions. Total mean lung lesion scores for vaccinated animals were significantly less (2 weeks old = 4.9; *p* = 0.0125 and 3 weeks vaccinated = 4.8; *p* = 0.0097) in comparison to 10.4 for the non-vaccinated control group at 10 days post challenge. Significantly less total mean lung lesions found in vaccinated pigs regardless of age of vaccination in comparison to non-vaccinated control pigs confirms the presence of protective, acquired immunity through vaccination.

### Viremia post challenge

Viremia was measured at necropsy (10 days post challenge) from lung tissue and serum samples and reported as log_10_ genome equivalence (GE)/ml (9). The frequency of positive piglets was 100% in the control group, whereas the proportions in the 2-wks and 3-wks vaccination groups were 90% and 96%, respectively. In the lung tissue the mean value of PRRSv RNA was 6.45 log_10_ GE/ml in the control group, while the 2-wks and 3-wks vaccination groups had mean values of 5.86 (*p* = 0.0449) and 6.22 (*p* = 0.2521), respectively. Serum samples collected at necropsy had a mean value of PRRSv RNA of 4.59 log_10_ GE/ml in the control groups and 3.76 (*p* = 0.0465) and 4.05 (*p* = 0.1357) in the 2-wks and 3-wks vaccination groups (Table 2).

**Table 2:** Frequency and viral load in lung tissue and serum at time of necropsy (10dpc).

Group	Frequency, positive animals [%]	Viremia in lung tissue		Viremia in serum sample	
		log_10_ GE/ml	*p*-value	log_10_ GE/ml	*p*-value
2-wks vaccination	90	5.86	0.0449	3.76	0.0465
3-wks vaccination	96	6.22	0.2521	4.05	0.1357
No vaccination	100	6.45		4.59	

### Transfer of maternal PRRSv-specific IgA and IgG antibodies from the dam to the piglet

**Fig 3:**
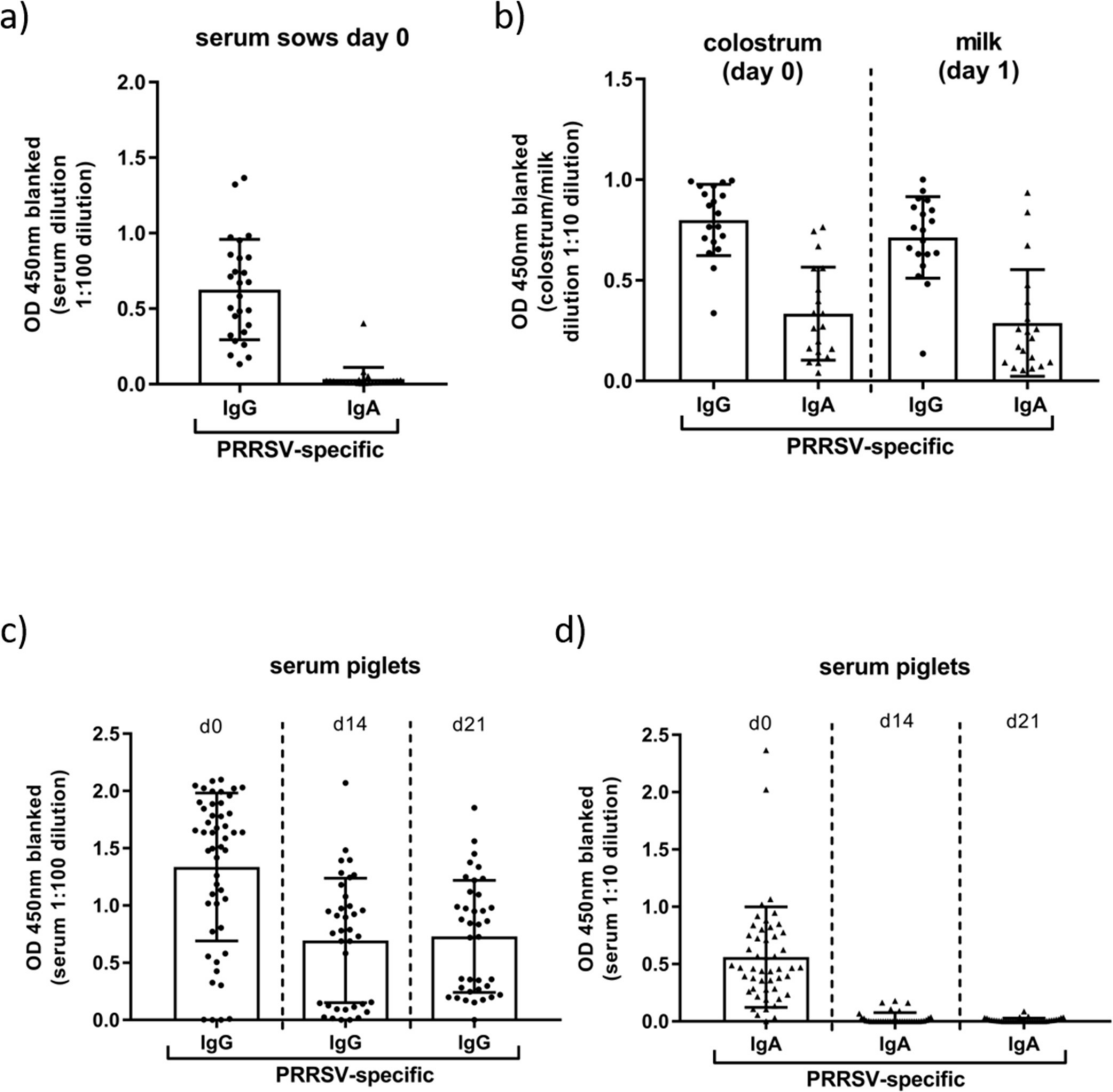
PRRSv-specifc IgG and IgA level in sows and piglets. a) Serum from sows was collected at the day of farrowing (day 0) and was analyzed for PRRSV-specific IgG and IgA levels. b) Colostrum and milk at the indicated time points (day 0 and day 1) after farrowing were collected and analyzed for PRRSv-specific IgG and IgA levels. Serum from piglets was taken at day 0, 14 and 21 and was analyzed for PRRSv-specific IgG (c) and IgA (d) levels.

To get a better impression of the transfer of maternal immunity from PRRS-vaccinated sows to the piglets, the level of PRRSv-specific IgA and IgG antibodies in sows and piglets were measured in serum at day of farrowing and in colostrum and milk (Fig 3A and 3B). The antibody levels showed a high variability in all tested fluids, however, PRRSv-specific IgG antibodies could be detected in serum, colostrum and milk without any significant difference between colostrum and milk among all vaccinated sows. In contrast, only a marginal level of serum IgA specific for PRRSv could be detected in the same serum dilution in vaccinated sows (Fig 3A). Moreover, PRRSv-specific IgA levels in colostrum and milk were similar for both, but lower in comparison to PRRSv-specific IgG levels. These data suggest that PRRS-MLV vaccination of sows during gestation led to an induction and secretion of PRRSv-specific IgG and IgA antibodies in colostrum and milk for maternal transfer to their offspring.

In line with data from milk and colostrum, the transfer of maternal IgA and IgG antibodies specific for PRRSv from the dam to the piglet could be shown in sera of piglets after colostrum intake post farrowing (Fig 3C and 3D). However, two and three weeks later decreased PRRSv-specific IgG antibody titers were detected with high variability among individual piglets (Fig 3C). Similar to sows, only marginal levels of PRRSv-specific IgA in serum were detected in piglets at two and three weeks of age compared to levels farrowing (Fig 3D). The detailed analysis of PRRSv-specific antibody levels in different specimens revealed that active immunization of pregnant sows with a PRRS-MLV lead to an induction of PRRSv-specific IgG and IgA antibodies, which were secreted to colostrum and milk and were transferred to suckling piglets. However, the majority of PRRSv-specific MDA, which were detectable in sera of piglets, consisted of IgG antibodies whereas the transferred PRRSv-specific IgA antibodies declined rapidly to undetectable levels.

### Colostrum and milk contain PRRSv-specific immune cells

**Fig 4:**
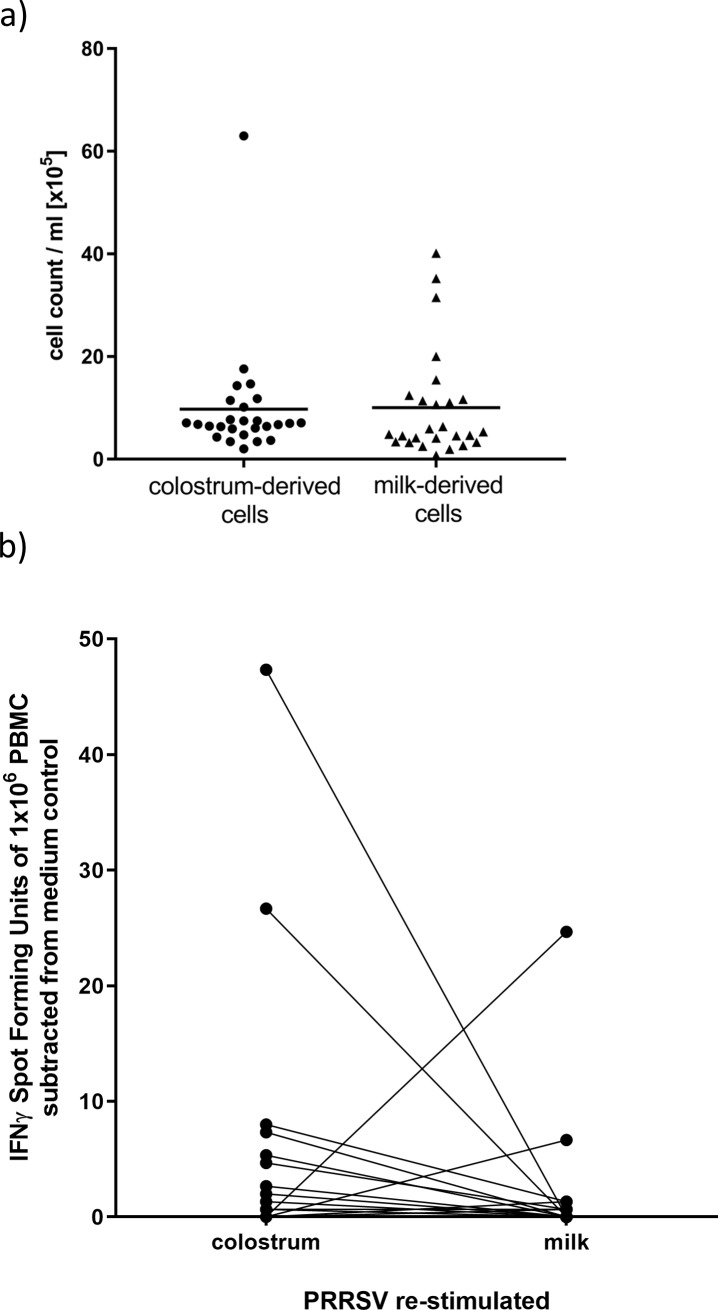
Cellular immunity in colostrum and milk. Cells were isolated from milk and colostrum and recalculated cell counts / ml were shown in a). b) Isolated cells from milk and colostrum were assessed for a PRRSv-specific IFNγ response by ELISpot. IFNγ spot forming units of milk and colostrum derived cells were stimulated either with medium or PRRSv virus (MOI 1.0). Each symbol represents the average spot forming units of 3 technical replicates for PRRS virus stimulation subtracted from the average of 2 technical replicates for medium stimulation, both performed on one sample from one individual animal. Samples of milk and colostrum from the same animal were connected with lines.

Total cells were isolated from colostrum and milk samples and analyzed for PRRSv-specific reactivity as part of the analysis of PRRSv-specific maternal immunity delivered from the dam to the piglet. The total cell count of colostrum- and milk-derived cells was assessed and no significant differences between both fluids (Fig 4A) were observed. In addition, cells isolated from colostrum and milk were stimulated with either PRRSv or growth medium as background control for 48h and then observed for PRRSv induced IFNγ production via IFNγ-ELISpot assay (Fig 4B).

The frequency of Spot Forming Units (SPU) showed high variability in responses between individual animals while some animals regardless of treatment did not show a measureable PRRSv specific IFNγ response. However, the majority of sows did transfer PRRSv specific IFNγ producing cells through colostrum and milk to the offspring. On average, the amount of IFNγ producing cells was higher in the colostrum-derived cell population than in the milk-derived cells (Fig 4B).

**Fig 5:**
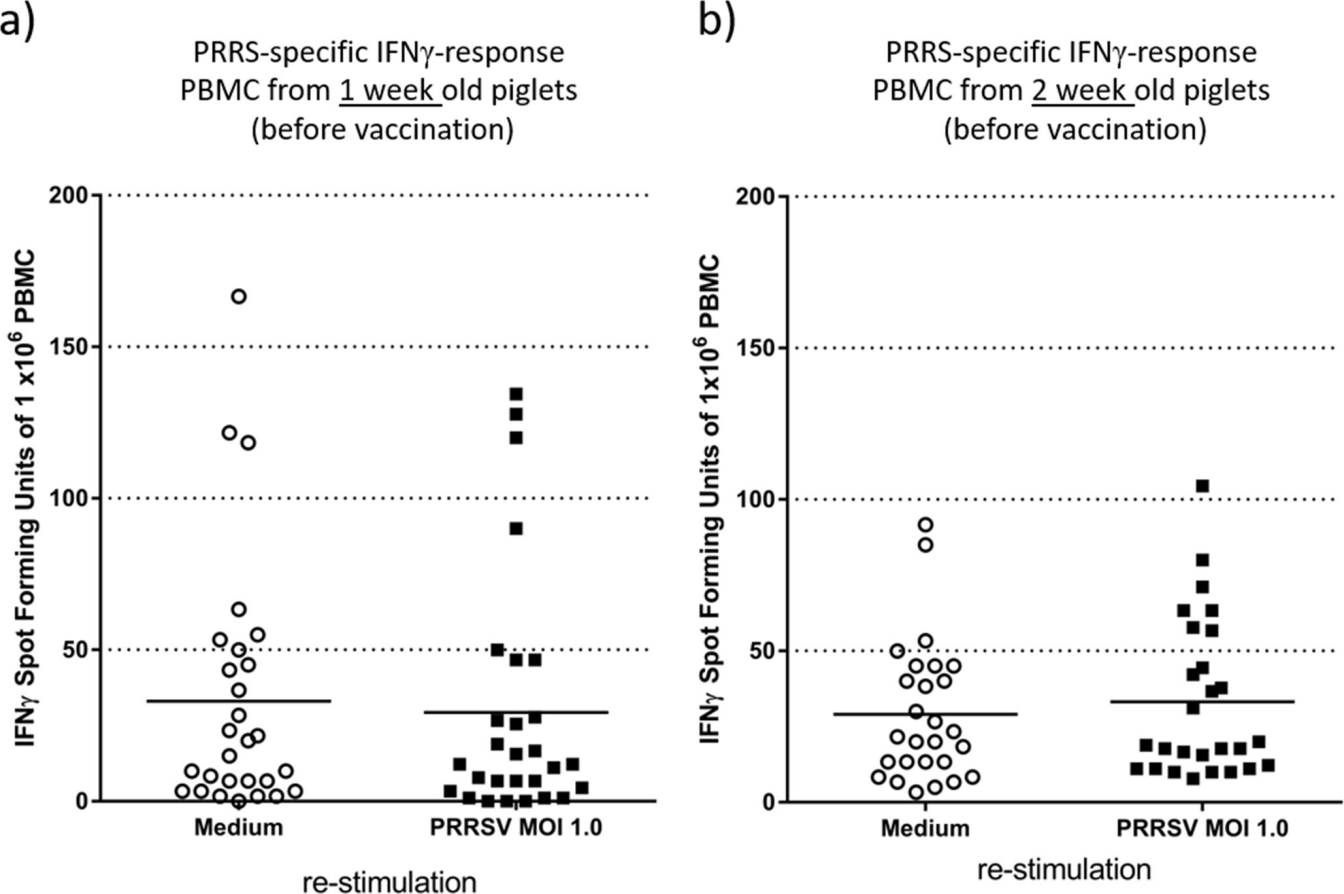
PRRSv-specific IFNγ response of PBMCs from 1- and 2-weeks old piglets. PBMCs derived from 1 week (a) and 2 week (b) old piglets were stimulated with medium and PRRS virus (MOI of 1.0) to determine the antigen-specific IFNγ response by ELISpot. Each symbol represents the average spot forming units of 3 technical replicates for PRRS virus stimulation and 2 technical replicates for medium stimulation, both performed on one sample from one individual animal. The horizontal bars represent the median spot forming units of 30 animals.

To investigate the transfer of maternally derived cells from the sow to the piglet the frequency of PRRSv-specific IFNγ producing cells in the blood of the piglet were analyzed via the IFNγ-ELISpot. The PBMCs from suckling piglets at one week and two weeks of age were isolated and stimulated for 48h with either PRRSv or with growth medium as background control. The majority of piglets showed no PRRSv specific IFNγ producing cells while some piglets did react to the PRRSv specific stimulation, both at one week and two weeks of life (Fig 5). However, PBMCs also cross-reacted to some extent to the mock stimulation making the IFNγ-ELISpot results difficult to analyze beyond descriptive statistics.

### Vaccination with Ingelvac PRRSFLEX^®^ EU induces a PRRSv-specific cellular and humoral immune response in the presence of maternal immunity

**Fig 6:**
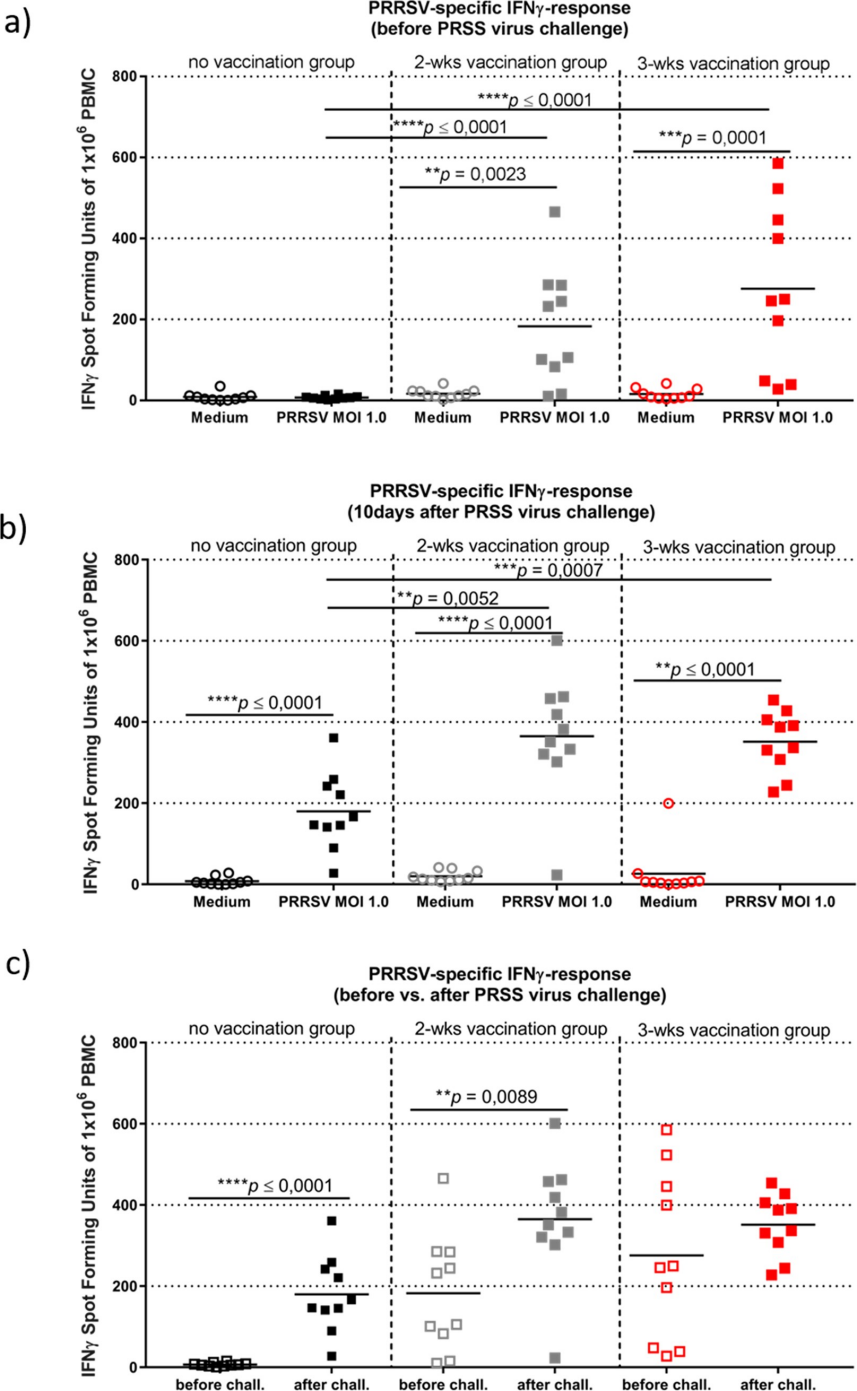
PRRSv-specific IFNγ response before and after challenge. Spot forming units of 1x10^6^ porcine PBMCs stimulated with RPMI medium (circles) or PRRS virus (squares, MOI of 1.0) were determined before PRRS virus challenge (a) and 10 days after PRRS virus challenge (b) by IFNγ ELISpot. c) Comparison of PRRSv (MOI 1) restimulated PBMCs before and after challenge for all three groups. Each symbol represents the average IFNγ-specific spot forming units of 3 technical replicates for PRRS virus stimulation and 2 technical replicates for RPMI medium stimulation, both performed on one sample from one individual animal. The horizontal bars represent the median spot forming units. Significant differences were calculated by Mann-Whitney-test and calculated *p*-values were indicated in the graph.

The significant reduction of lung lesions after challenge (Fig 2) together with PRRSv-specific antibody screens in the vaccinated groups suggested a successful vaccination in the presence of MDA. For a more detailed analysis, blood was taken from piglets before and after challenge to precisely analyze the PRRSv-specific cellular and humoral immune responses related to vaccination-only or vaccination-challenge events. First, PBMCs and sera were extracted from blood from all groups at nine weeks of age (one week prior to challenge), and at necropsy (ten days after challenge). Isolated PBMCs were used at a concentration of 3x10^5^ PBMCs/well and were stimulated with either PRRSv at a multiplicity of infection (MOI) of 1.0, or with growth medium as control to assess the PRRSv-specific IFNγ response (Fig 6A and 6B).

The ELISpot data demonstrate that vaccinated piglets showed a significant immunological reaction to PRRSv specific stimulation in terms of IFNγ producing PBMCs for both vaccinated groups compared to the non-vaccinated group at six to seven weeks post vaccination (one week before challenge) (Fig 6A). The overall number of cells showing reactivity to PRRSv or mock stimulation was highly variable among individual animals. However, the mean number of spot forming units (SFU) was the highest for the group vaccinated at 3 weeks of age with an average of 245 SFU per 1x10^6^ PBMCs compared to an average of 170 SFU per 1x10^6^ PBMCs in the group vaccinated at 2 weeks of age. The non-vaccinated control group did not show an increase in IFNγ secreting PBMCs upon stimulation with PRRSv at all, confirming that this group was not exposed to either field virus or vaccine. Differences between vaccinated groups and non-vaccinated animals were highly significant (*p* ≤ 0.0001).

At necropsy, ten days after challenge with a heterologous PRRSv, the number of IFNγ producing PBMCs for all groups increased compared to their IFNγ levels before challenge. Additionally, individual animal variability in the vaccinated groups was greatly reduced. However, only the non-vaccinated, control group and the 2-wks vaccination group had IFNγ levels increased significantly (*p* ≤ 0.0001 and *p* = 0.0089) (Fig 6C). The group vaccinated at 3 weeks of age did not differ from the group vaccinated at 2 weeks of age, with median values of 366 SFU and 362 SFU, respectively (Fig 6B and 6C). After exposure to the PRRS challenge virus, the non-vaccinated, control group showed a mean value of 156 SFU per 1x10^6^ PBMCs (Fig 6B). Overall, differences between vaccinated groups and non-vaccinated animals remained to be highly significant (*p* = 0.0007 and *p* = 0.0052) (Fig 6B).

**Fig 7:**
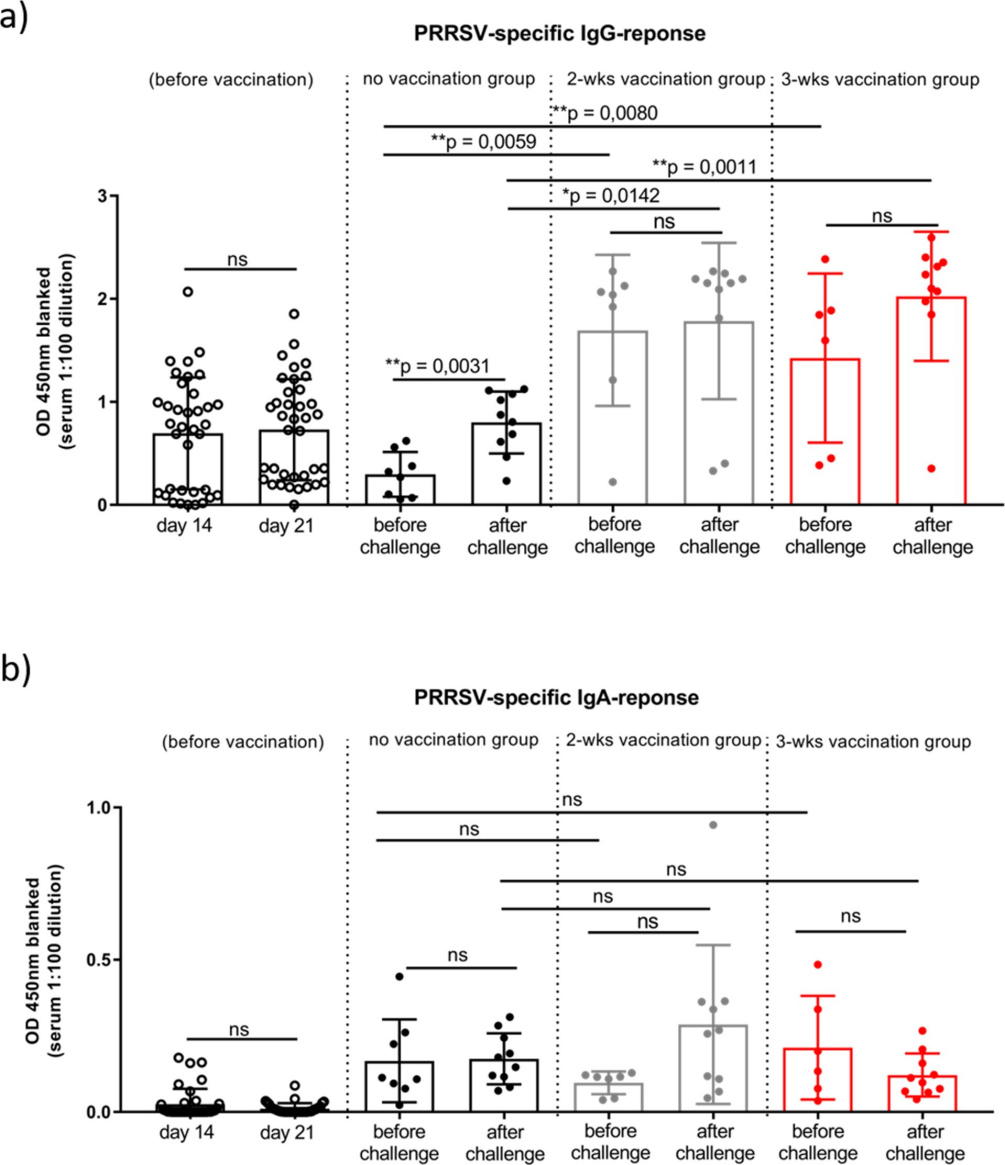
PRRSv-specific antibody response before and after challenge. a) PRRSv-specific IgG and (b) IgA response were assessed by PRRSv-specific ELISA at the indicated time points. Each symbol represents the calculated average of one animal. Significant differences were calculated by Mann-Whitney-test and calculated *p*-values were indicated in the graph.

Analysis of the PRRSv-specific humoral immune response after vaccination and challenge in more detail revealed a similar picture. Whereas, the PRRSv-specific IgA levels in sera did not show any significant differences between the groups before or after challenge (Fig 7B) PRRSv-specific IgG levels significantly increased after vaccination in both vaccinated groups (*p* = 0.008 and *p* = 0.0059) compared to the non-vaccinated, control group (Fig 7A). Ten days after the heterologous PRRSv challenge, the PRRSv-specific IgG levels increased significantly in the non-vaccinated, control group (*p* = 0.0031) whereas, the vaccinated groups showed no further increase in PRRSv-specific IgG titers. However, the PRRSv-specific IgG levels of both vaccinated groups were still significant higher overall compared to the non-vaccinated animals (*p* = 0.0142 and *p* = 0.0011) (Fig 7A).

### Vaccination reduce the pulmonary level of pro-inflammatory cytokines after PRRSV challenge

**Fig 8:**
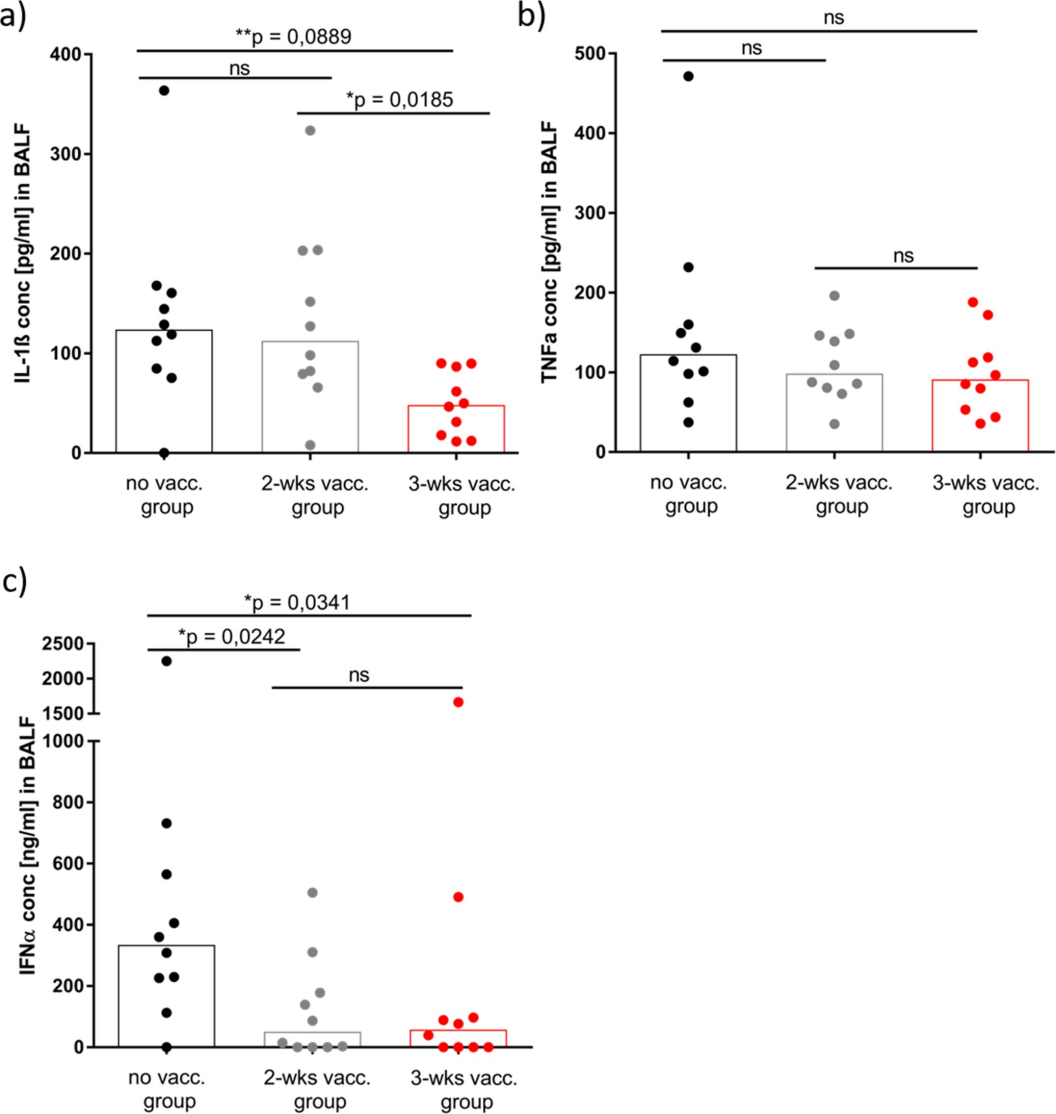
Pro-inflammatory cytokine response 10 days after PRRS virus challenge from BALF. 10 days after PRRS virus challenge BALF were taken at necropsy. Concentrations of the cytokines IL-1β (a), TNFα (b) and IFNα (c) were determined by ELISA. Each symbol represents the average of 2 technical replicates of BALF samples from one individual animal. The bars represent the median concentration in pg/ml (IL-1β and TNFα) and ng/ml (IFNα) of 10 animals analyzed from each respective group. Significant differences were calculated by Mann-Whitney-test and calculated *p*-values were indicated in the graph.

In line with the pathological findings, levels of pulmonary cytokine were assessed among animals in all groups in this study (Fig 8). The pro-inflammatory cytokines IL-1ß, TNFα and IFNα were analyzed in the BALF of all animals at necropsy (10 days after challenge). The pro-inflammatory cytokine IL-1β was significantly reduced in the 3-wks vaccination group (48pg/ml) in comparison to the 2-wks vaccination group (112pg/ml; *p* = 0.0185) and challenge control group (124pg/ml; *p* = 0.0089) (Fig 8A). In contrast to this finding, the pro-inflammatory cytokine TNFα showed no significant reduction in the two vaccinated groups compared to the challenge control group (Fig 8B). Notably, both vaccinated groups showed a significantly reduced IFNα concentration (50pg/ml and 57pg/ml) in the BALF in comparison to the challenged non-vaccinated control group (334.5pg/ml; *p* = 0.0341 and *p* = 0.0242) (Fig 8C).

## Discussion

The potential for interference in the successful vaccination of young piglets from maternally derived antibodies poses a significant challenge for the global swine industry. The demand for early protection against clinical disease and the associated economic losses coupled with current standard husbandry practices provide an incentive to vaccinate piglets as early as possible. The transfer of maternally derived antibodies from sow to piglet has been shown to interfere with the antibody response to PRRSv vaccination [19, 29] or Swine influenza virus vaccination [30]. However recently, it was demonstrated in a field experiment that a PRRSv vaccine was able to overcome the maternally derived antibody interference and protected piglets vaccinated at two and three weeks of life from a field challenge [21]. Moreover, a recent publication could show the vaccine efficacy of a MLV PRRSv vaccine in 1-day-old piglets in the presence of transferred maternally derived PRRSv specific antibodies [22]. Based on these findings, it was attempted to characterize different immunological parameters at different time points in a similar approach. In this study, two groups of piglets vaccinated at two weeks and three weeks of age in the presence of maternally derived antibodies against a homologous PRRSv strain to the vaccine were analyzed for lack of interference to vaccine efficacy in comparison to a non-vaccinated, control group. All groups were challenged with a heterologous PRRSv to assess the protective effect of vaccination and evaluate the immune response and associated correlates to vaccine efficacy. The time point of challenge was chosen to allow the challenge control group to be waned of MDA and to function as a true negative control group. However, a group with no MDA at vaccination would have been of interest to analyze side-by-side. Due to the study design of a field-based vaccination, this was not feasible; including a naïve group from a different source would have altered the results through a different genetic background and environmental conditions. Further research studies need to be conducted to evaluate this aspect.

The early protection of newborns against pathogens by transferred maternal immunity from the mother to the offspring is a well-accepted mechanism. In swine, colostrum intake by piglets is well-known to be beneficial for the development of piglets. Transfer of immunoglobulins and cells of the dams immune system to the piglet through colostrum and milk to protect the piglet from early diseases is well described [13]. In this study, cell counts in colostrum and milk revealed sows effectively secreted and transferred immune cells to the piglet. More specifically, some dams transferred PRRSv-specific cells to the piglet with higher numbers in colostrum than in milk. In addition, colostrum and milk contained PRRSv-specific IgA and IgG levels that may have added to protection of the newborn piglet against PRRSv infection. Despite the fact that PRRSv-specific cells were detected and transferred in colostrum and milk to piglets, analysis of PRRSv-specific cells retrieved from blood of the piglets at one or two weeks of age remained questionable. It is plausible that maternally derived cells were diminished due to duration of time from suckling to vaccination or were below the detection limit of the test. Nevertheless, PRRSv-specific IgG antibodies in sera could be detected until weaning (four weeks of age) when the titer decreased under the test’s detection limit in animals from the non-vaccinated group. These findings indicated a stable, transferable, anti-PRRSv specific humoral immunity from the mother to the piglet during the first weeks of life until weaning. These findings also demonstrated the need for vaccination to fill in the gap between protective maternal immunity and a sufficient piglet derived immune response against potential pathogens.

Despite the stable, maternally derived anti-PRRSv-specific IgG levels in the piglets, data presented in this study suggests that vaccination as early as two weeks of age led to efficient humoral and cellular immune responses against PRRSv in the presence of MDA. Acquired cellular immunity was detectable in piglets after vaccination at 9 weeks of age (6–7 weeks post vaccination) in both vaccinated groups regarding PRRS virus specific IFNγ-responses. The non-vaccinated control group showed no IFNγ-response to PRRS virus stimulation. These findings indicate a clear vaccine dependent cellular immune response was achieved when vaccinating in the presence of maternal immunity at 2–3 weeks of age.

After challenge, the IFNγ-response got more robust in vaccinated animals (booster effect) while non-vaccinated animals developed a cell mediated response at levels indicative of a primary response to PRRSv infection. The differences between vaccinated and non-vaccinated control groups were highly significant (*p* = 0.0007 and *p* = 0.0052). Additionally, before challenge the number of PBMCs producing IFNγ upon *in vitro* stimulation with PRRSv was slightly higher for those animals vaccinated at three weeks of age compared with the animals vaccinated at two weeks of age. However, differences in IFNγ production between vaccinated groups were diminished 10 days after challenge.

In regards to the vaccine-induced humoral PRRSv-specific immune response, significant differences were detected for IgG before and after challenge for the vaccinated groups in comparison to the non-vaccinated group (p<0.0044). Interestingly, the PRRSv-specific IgG titers were not increased after challenge in the 2- and 3-wks vaccination group in contrast to the booster effect observed the acquired cellular immunity in these vaccinated animals. Perhaps, this was due to the intranasal challenge which can trigger a mucosal dominated IgA stimulation and to a lesser extent, a systemic IgG serological response.

Regardless, a primary humoral response was observed at ten days post challenge for the non-vaccinated group with a significant increase in PRRSv-specific IgG antibodies.

This study represented a worst-case scenario concerning maternally derived antibodies. Since the sows were vaccinated with a homologous PRRSv strain identical to the vaccine strain of the piglets, a higher probability of vaccine interference was expected. The mechanisms of maternally derived antibody interference are still elusive. Transferred maternal neutralizing antibodies (NA) are capable to interfere with vaccination. However, even non-neutralizing antibodies are able to interfere with vaccination, too (summarized in [31]). Heterologous PRRSv strains can induce different levels of neutralizing antibodies (NA); however, vaccine strains tend to induce only low levels of NA [32, 33]. Since the dams were vaccinated, only minor levels of NA in sows and an even more reduced level of transferred NA by colostrum and milk might be expected. Therefore, it is rather unlikely that transferred NA by vaccinated sows to piglets have major interfering effects on piglet vaccination. In consequence, only PRRSv-specific antibody levels were analyzed in this study without further differentiation between neutralizing and non-neutralizing antibodies. Nevertheless, our data demonstrate that even in the presence of potentially transferred homologous NA by the dam an immune reaction in the offspring after vaccination was not inhibited since the decline of PRRS specific (general) serum antibodies was reversed over time, which underlines vaccine efficacy.

The production of pro-inflammatory cytokines in PRRSv infections was described to be involved in the induction of tissue lesions [34]. Acute infection with wild type PRRSv suppresses the production of IFNα in the lungs by large [35] [36]. In this study, animals showed a significantly decreased production of IFNα, compared to the non-vaccinated animals post challenge. More research needs to be conducted to completely understand the reduction of IFNα in vaccinated animals. We hypothesize that this reduction reflected the decreased viral load in lung tissue and serum due to the amnestic response and likely played an important role in the reduction of lung tissue damage in vaccinated animals. In addition, the reduction in lung damage may aid in the suppression of secondary infections of opportunistic pathogens under field conditions and therefore, suggests positive indirect effects of vaccination.

Ultimately, the most important parameter to assess the differences in response between vaccinated and non-vaccinated animals was the presence of lung lesions due to PRRSv challenge. Damaged lung tissue is the driving force of some of the clinical signs such as dyspnea and dermal cyanosis [37]. In addition, affected tissue is vulnerable to infection with other bacterial pathogens that negatively enhance the clinical outcome [38] [39]. In this study, animals were challenged at 10 weeks of life at approximately seven to eight weeks post vaccination. Both vaccinated groups showed a significant reduction of lung lesions compared to the non-vaccinated (p<0.0125), but challenged group. This result not only confirms the efficacy of the vaccination in the presence of maternal immunity, it also suggests a lower risk of secondary infections in a field situation. Further clinical parameters like rectal temperature and weight gain were investigated, however, due to the shortened post challenge period, no consistent statistical difference could be detected in these parameters.

In conclusion, vaccination with Ingelvac PRRSFLEX^®^ EU in the presence of maternal immunity effectively induced both, a humoral and cellular immune response to a PRRSv challenge and significantly reduced the clinical outcome of infection under controlled challenge conditions.
